# Ultrastructural changes in esophageal tissue undergoing stretch tests with possible impact on tissue engineering and long gap esophageal repairs performed under tension

**DOI:** 10.1038/s41598-023-28894-5

**Published:** 2023-01-31

**Authors:** Ede Biro, Gerhard Sommer, Gerd Leitinger, Hajnalka Abraham, Daniel J. Kardos, Zsolt Oberritter, Amulya K. Saxena

**Affiliations:** 1 Department of Paediatrics, Division of Paediatric Surgery, University of Pécs Medical School, Jozsef Attila St. 7, Pécs, 7634 Hungary; 2 Institute of Biomechanics, Graz University of Technology, Graz, Austria; 3 Research Unit Electron Microscopic Techniques, Division of Cell Biology, Histology and Embryology, Gottfried Schatz Research Center, Medical University of Graz, Graz, Austria; 4 Department of Medical Biology and Central Electron Microscopic Laboratory, University of Pécs Medical School, Pécs, Hungary; 5 Department of Pediatric Surgery, Chelsea Children’s Hospital, Chelsea and Westminster Hospital NHS Fdn Trust, Imperial College London, 369 Fulham Road, London, SW10 9NH UK

**Keywords:** Gastroenterology, Gastrointestinal diseases, Biomedical engineering, Oesophageal diseases

## Abstract

Esophageal biomechanical studies are being performed to understand structural changes resulting from stretches during repair of esophageal atresias as well as to obtain biomechanical values for tissue-engineered esophagus. The present study offers insights into ultrastructural changes after stretching of the ovine esophagus using uniaxial stretch tests. In vitro uniaxial stretching was performed on esophagi (*n* = 16) obtained from the abattoir within 4–6 h of 1-month-old lambs. Esophagi were divided into 4 groups (4 esophagi/group): control, Group1 (G1), Group2 (G2), Group3 (G3) stretched to 20%, 30% and 40% of their original length respectively. Force and lengthening were measured with 5 cycles performed on every specimen. Transmission electron microscopic (TEM) studies were performed on the 4 groups. During observational TEM study of the control group there were no significant differences in muscle cell structure or extracellular matrix. In all stretched groups varying degrees of alterations were identified. The degree of damage correlated linearly with the increasing level of stretch. Distance between the cells showed significant difference between the groups (control (μ = 0.41 μm, SD = 0.26), G1 (μ = 1.36 μm, SD = 1.21), G2 (μ = 2.8 μm, SD = 1.83), and G3 (μ = 3.01 μm, SD = 2.06). The diameter of the cells (control μ = 19.87 μm, SD = 3.81; G1 μ = 20.38 μm, SD = 4.45; G2 μ = 21.7 μm, SD = 6.58; G3 μ = 24.48 μm, SD = 6.69) and the distance between myofibrils (control μ = 0.23 μm, SD = 0.08; G1 μ = 0.27 μm, SD = 0.08; G2 μ = 0.4 μm, SD = 0.15; G3 μ = 0.61 μm, SD = 0.2) were significantly different as well ( *p* < 0.05 was considered to be significant). Esophageal stretching > 30% alters the regular intracellular and extracellular structure of the esophageal muscle and leads to disruption of intra- and extracellular bonds. These findings could provide valuable insights into alterations in the microscopic structure of the esophagus in esophageal atresias repaired under tension as well as the basis for mechanical characterization for tissue engineering of the esophagus.

## Introduction

Esophageal atresia is a congenital malformation occurring in 1:3000–5000 births^1^ consist of partial esophageal absences. Successful repairs were initially performed as staged procedures in 1939; with the first successful primary repair in 1941^2^. Improvement in survival increased from < 60% in the 1950′s to > 90% by the mid-1980’s^3^. Long-term follow-ups have indicated that patients after primary repair exhibit long-term morbidities^4, 5^. In neonates with long-gap atresia, delayed or staged repair, myotomy, and esophageal replacement with gastrointestinal transposition have been employed successfully to bridge the gap^6–8^. Unfortunately, these procedures are associated with a high rate of short and long-term complications^9, 10^. Hence, there is a demand for tissue-engineered esophagus to overcome this specific tissue shortage.

Our group has investigated the options in engineering an experimental ovine model, with success in (a) in vitro generation of esophageal constructs (epithelial cells and smooth muscle cells)^11–14^, and (b) the *in-situ* engineering of rudimentary esophageal tissue^15^. For that, it was important to obtain parameters on the biomechanics of the native esophagus to serve as an in vivo comparison to the tissue-engineered one in the future^16^. At the same time, the esophageal specimens that underwent controlled biomechanical stretch tests were evaluated by the same group histologically to provide information on possible structural changes that could explain esophageal morbidity such as anastomotic stricture and dysmotility after atresia repairs^16^.

The aim of the present study was to perform *in-vitro* controlled uniaxial stretch tests on 1-month-old lamb esophagus and also to investigate the effect of the elongation on the esophageal muscle tissue (which is the very similar maneuver done during esophageal atresia repair) using transmission electron microscopy (TEM). Ultrastructural changes were compared with varying degrees of stretch.

## Materials and methods

### Preparation of the specimens and stretch tests

Ovine esophagi from 1-month-old lambs were obtained from the local abattoir and transported on ice to the laboratory for the commencement of the investigations within 4–6 h. All experiments were conducted under the guidelines of the Committee for Animal Research, Federal Ministry of Science and Research, Vienna, Austria.

**Figure 1:**
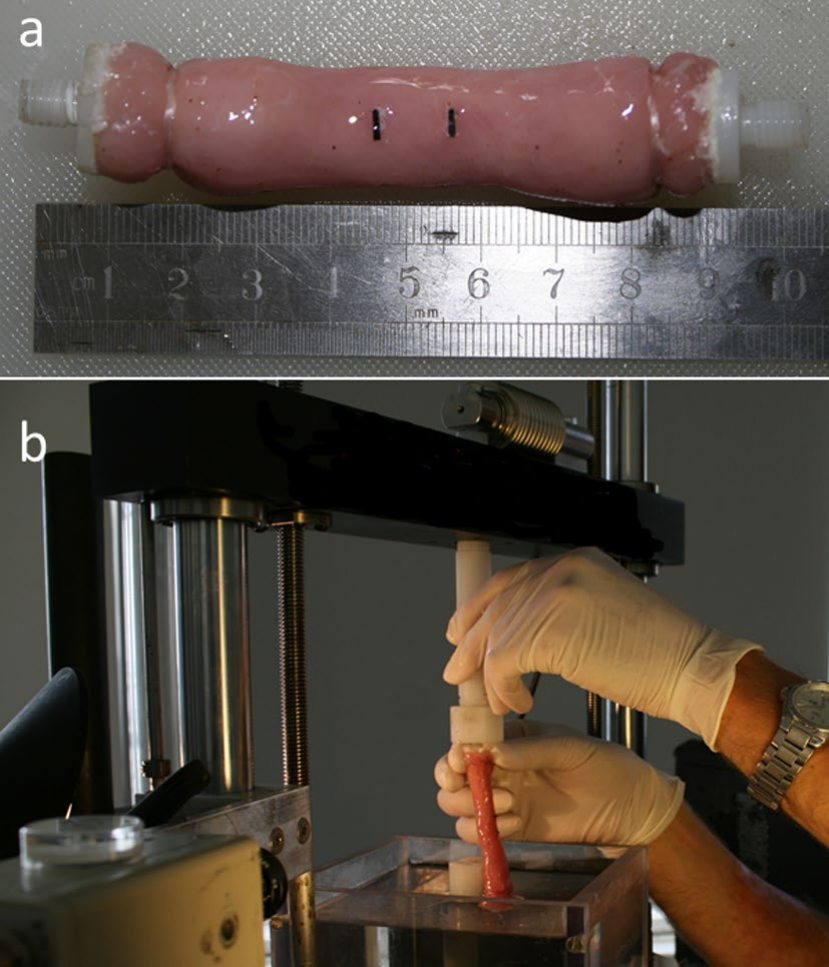
Experimental setup. (**a**) Mid-sections of the esophagus was investigated with gage markers glued for video extensometer detection. (**b**) The ends were secured with yarns to plastic screws for insertion in the equipment. The esophagus was immersed in a Perspex container filled with PBS at body temperature.

The methodology of the stretch tests remained similar to our previously published research (Fig. 1)^16^. The esophagi were divided into 4 groups (4 esophagi/group): control, Group 1 (G1), Group 2 (G2), Group 3 (G3) stretched to 20%, 30% and 40% of their original length respectively. On arrival in the laboratory, the esophagus was rinsed with PBS to remove any contents within the lumen. The middle 70–80 mm portion of the esophagus was prepared and utilized for the investigations (Fig. 1a). The ends of the esophagus were secured with yarns to the plastic screws of the strain instrument. Gage markers for the length measurements via the video extensometer were glued to the esophagus 1 cm apart in the mid portion of the investigated segment. The uniaxial stretch test experiments were performed on a computer-controlled screw-driven high precision tensile/compressive testing machine adapted for small biological specimens (μ-Strain Instrument ME 30–1, Messphysik) (Fig. 1b).

The specimens were investigated in a Perspex container filled with PBS solution (pH 7.3 containing 100 mg/l EDTA) maintained at 37 ± 0.1 °C by a heater-circulation unit (type Ecoline E 200, Lauda; Lauda-Königshofen, Germany) The tensile force was measured with a 25 N class 1 strain gage-load cell (model F1/25 N, AEP converter)^17^. The upper and lower crossheads of the testing machine are moving in opposite directions to enable the gage region of the samples in the constant field of view. A crosshead stroke resolution of 0.04 μm and a minimum load resolution of 1 mN of the 25 N load cell were employed. Digital control of the electric drive of the machine as well as data acquisition of the crosshead position and the applied load was done by an external digital controller (EDC 5/90 W, DOLI; Munich, Germany). For automatic gage mark and edge recognition a PC-based (CPU 586) video extensometer (model ME 46–350, Messphysik) was used^17, 18^. The experiments were conducted at 10 mm/min with samples subjected to 5 cycles each to achieve the desired stretch per group.

### Electron microscopic investigation

After completion of the stretch experiments, all esophagi from all groups were sampled multiple times and locations. The harvested 1–2 mm^3^ muscular layer samples 4 groups were fixed in 2.45% glutaraldehyde and 2.45% paraformaldehyde in a 0.1 M sodium cacodylate buffer (pH = 7.4) at room temperature for 4 h and at 4 °C for 16 h. The samples were washed in a 0.1 M sodium cacodylate buffer (pH = 7.4) at room temperature for 3 h and then post-fixed with 2% osmium tetra oxide at room temperature for 2 h. Afterwards the tissue was dehydrated in graded series of ethanol (50%, 70%, 90%, 96%, and 100%, each for 30 min at room temperature) and embedded in TAAB epoxy resin (TAAB Laboratories Equipment Ltd., UK). To determine the range of interest of the embedded samples, semi-thin Sects. (500 nm) of the embedded tissue was stained with 0.5% toluidine blue (FLUKA, Lot: BCBL 8513 V) in an aqueous solution and analyzed with light microscopy (Nikon Eclipse E800, equipped with a Nikon DN100 camera). For transmission electron microscopy (TEM), ultra-thin sections were prepared (70 nm) with ultramikrotom (LEICA, UC6, Wetzlar, Germany) and transferred on to copper grids. Additionally, they were stained with uranyl acetate (TED PELLA Inc., Lot: CA-1206) and lead citrate (DELTA MICROSCOPIES, Lot: REY 2618). For transmission electron microscopy FEI Tecnai G2 20 (Hillsboro, Oregon, United States) was used, equipped with CCD camera (Gatan US1000, Gatan, Pleasanton, USA) to obtain pictures. For image post-processing and measurements i-Team V10.1 (Olympus Soft Imaging Solutions GmbH, Technologiepark, Münster, Germany) was used.

### Quantification analysis

**Figure 2:**
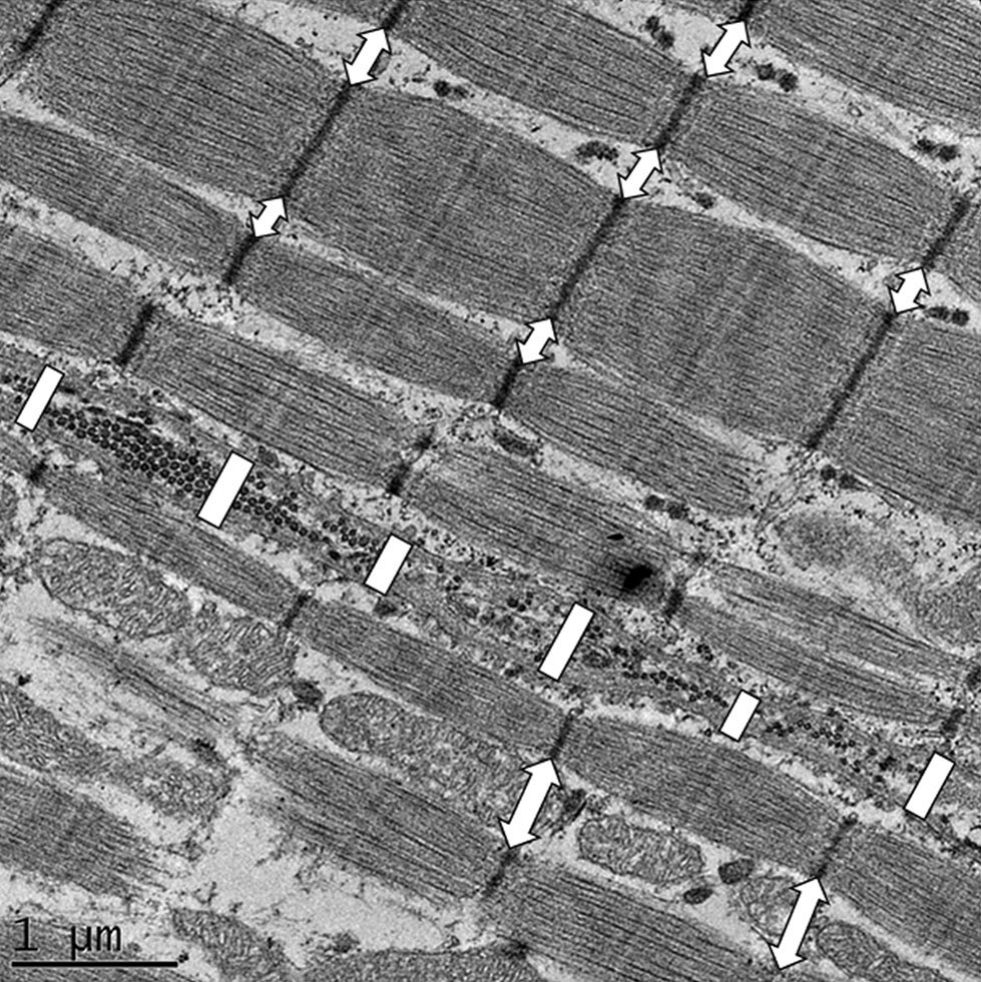
TEM measurements method. Electron microscopic image showing measured distance between cells (lines) and myofibrils (double arrows); distance of phospholipid membranes measured in 1–2 μm gradients and at the level of Z lines of sarcomeres.

To compare and quantify the ultrastructural changes in the tissues the following measurements were done in all 4 groups. To objectively determine the severity of intracellular damage, correlations between stretch levels and the diameter of the muscle cells as well as distance between myofibrils in the muscle cells were analyzed. To quantify the effect of mechanical stress on to extracellular matrix and connections between muscle cells the distance between cell membranes lying next each other was measured (Fig. 2). Previous three morphological parameters were measured in all groups as many times as possible on every sample (average number of measurements done per groups: Distance between muscle cells N = 299, Diameter of the muscle cells N = 213, Distance between myofibrils N = 970).

For statistical analysis IBM SPSS Statistics 27.0.1 (Armonk, New York, USA) was used. Continuous data were tested for normality and presented in means and standard deviations. Outliers were interpreted as measurement errors and excluded. Independent-Samples Kruskal–Wallis test was used to compare groups. The null hypothesis was that the distribution of measured distances is the same across the 4 Groups. A *p* < 0.05 was considered to be significant. Significance values have been adjusted by the Bonferroni correction for multiple tests.

## Results

### Morphological (qualitative) TEM results:

**Figure 3:**
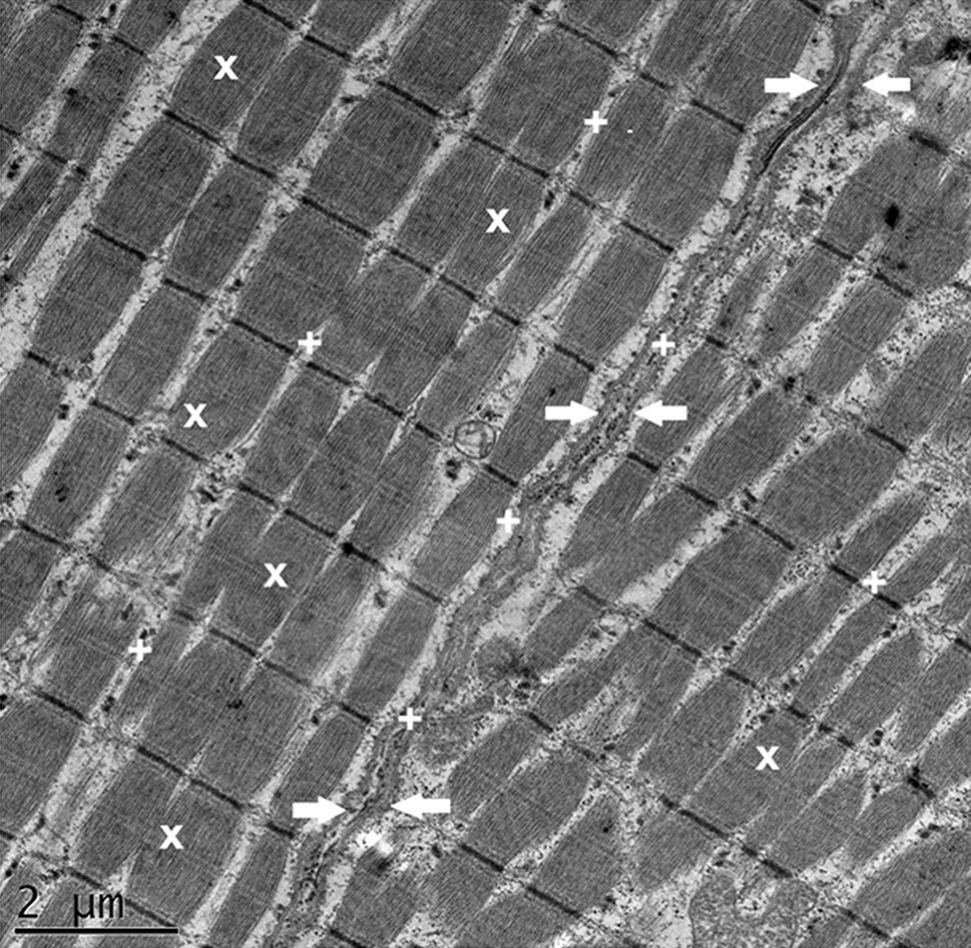
Control group (normal). TEM image demonstrates preserved myofibrillar structure (x), normal distance between approximating cells phospholipid membranes (arrows), intact intracellular and extracellular spaces (+).

During observational study of the control group, there were no significant differences in muscle cell structure or extracellular matrix (Fig. 3).

**Figure 4:**
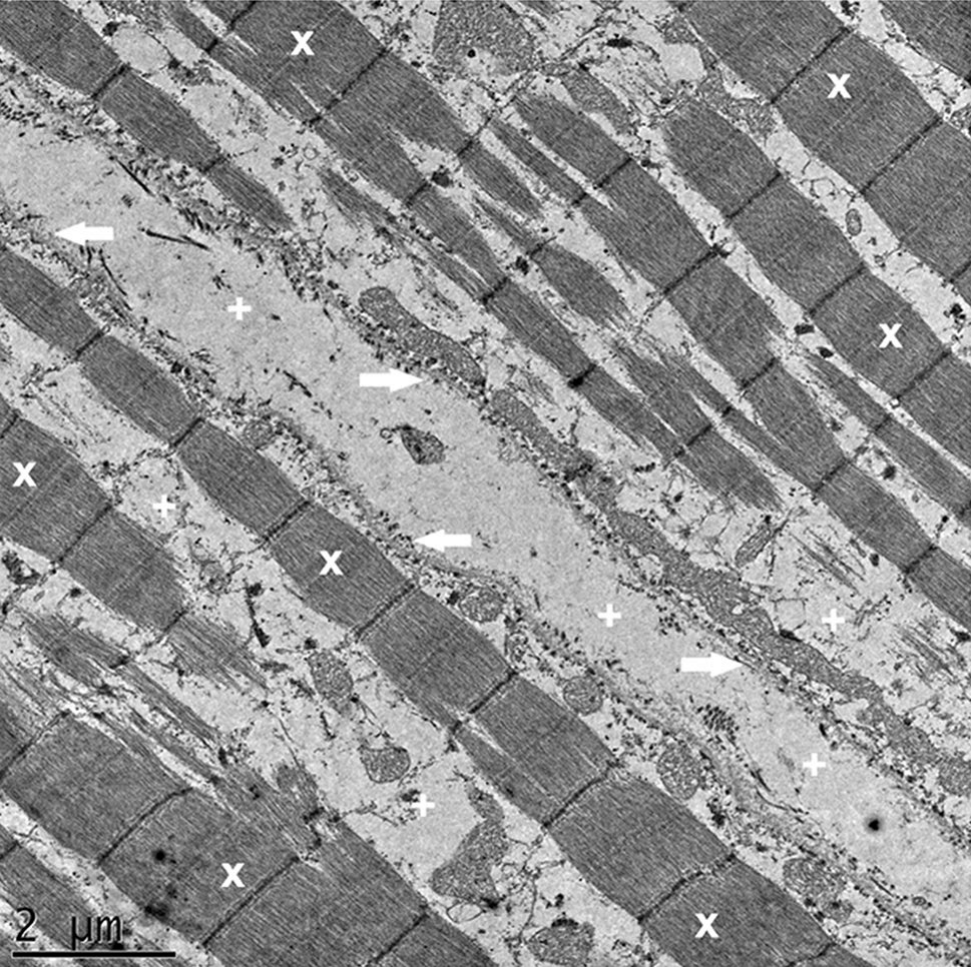
Group1 (G1, stretched to 20%). Stretch G1 TEM image demonstrating preservation of majority of sarcomers and identifiable myofibrillar structure (X); however, the distance between the myofibrils, and the cells is increased (+), with the cell membrane still attached to the intracellular structures (arrows).

**Figure 5:**
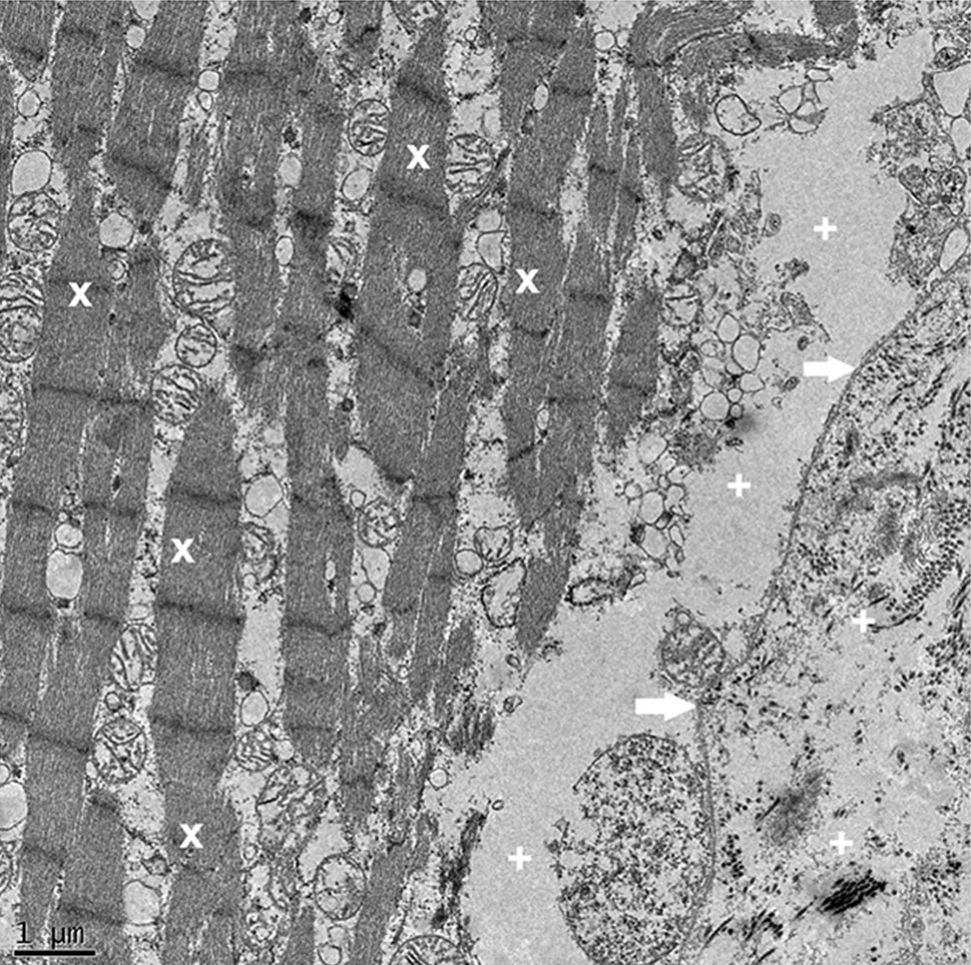
Group3 (G3, stretched to 40%). Stretch G3 TEM image demonstrating anomalous myofibrillar structure (X), abnormal gap between the myofibrills and the cell membrane, enlarged extracellular space (+), and cell membrane disruption from the intracellular structures (*arrows*).

In the three stretched groups (G1, G2, G3) varying degrees of alterations were found in the samples. The degree of damage progressed with the increasing level of stretch (Figs. 4, 5). In G1 myofibrillar structure was preserved. However, the distance between the myofibrils was increased compared to control group. Furthermore, in G1 the cell membrane attachment to its intracellular matrix remained normal, but connections between cells were disrupted. In G2 beside the discrepancies mentioned above in G1, myofibrils and structure of the sarcomeres were found to be disrupted as well. All these ultrastructural damages were even more prominent in G3.

Based on observed morphological changes and to demonstrate objectively the structural tissue damages, the following three parameters were measured: a. Distance between muscle cells, b. Diameter of the muscle cells, c. Distance between myofibrils.

### Quantitative TEM results:

#### Distance between muscle cells

**Figure 6:**
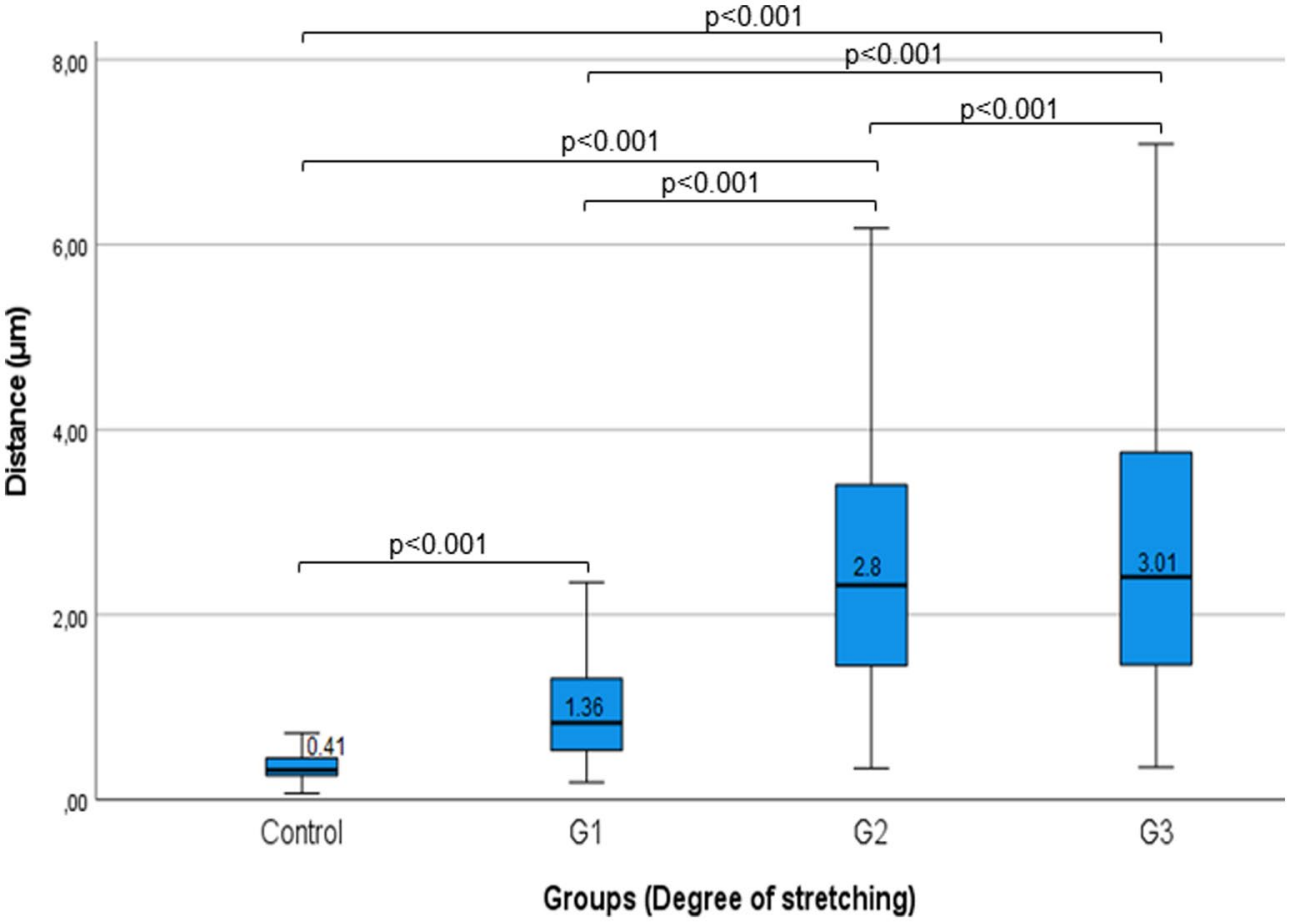
Distance between muscle cells. Boxplot diagram showing the measured distance between cells in the four Groups and the observed significant difference by paired comparisons of the Groups.

Normality test showed abnormal distribution of the measured data. Significant difference in cell distance was measured between the control (μ = 0.41 μm, SD = 0.26), G1 (μ = 1.36 μm, SD = 1.21), G2 (μ = 2.8 μm, SD = 1.83), and G3 (μ = 3.01 μm, SD = 2.06) groups. Only G2 and G3 were found to have the same extremely high level of cell disruption (Fig. 6). The mean distance increased parallel to applied stretch level. *p* values are < 0.001 as well in case of calculating the adjusted significance values by the Bonferroni correction, that indicate even stronger significance.

#### Diameter of the muscle cells

**Figure 7:**
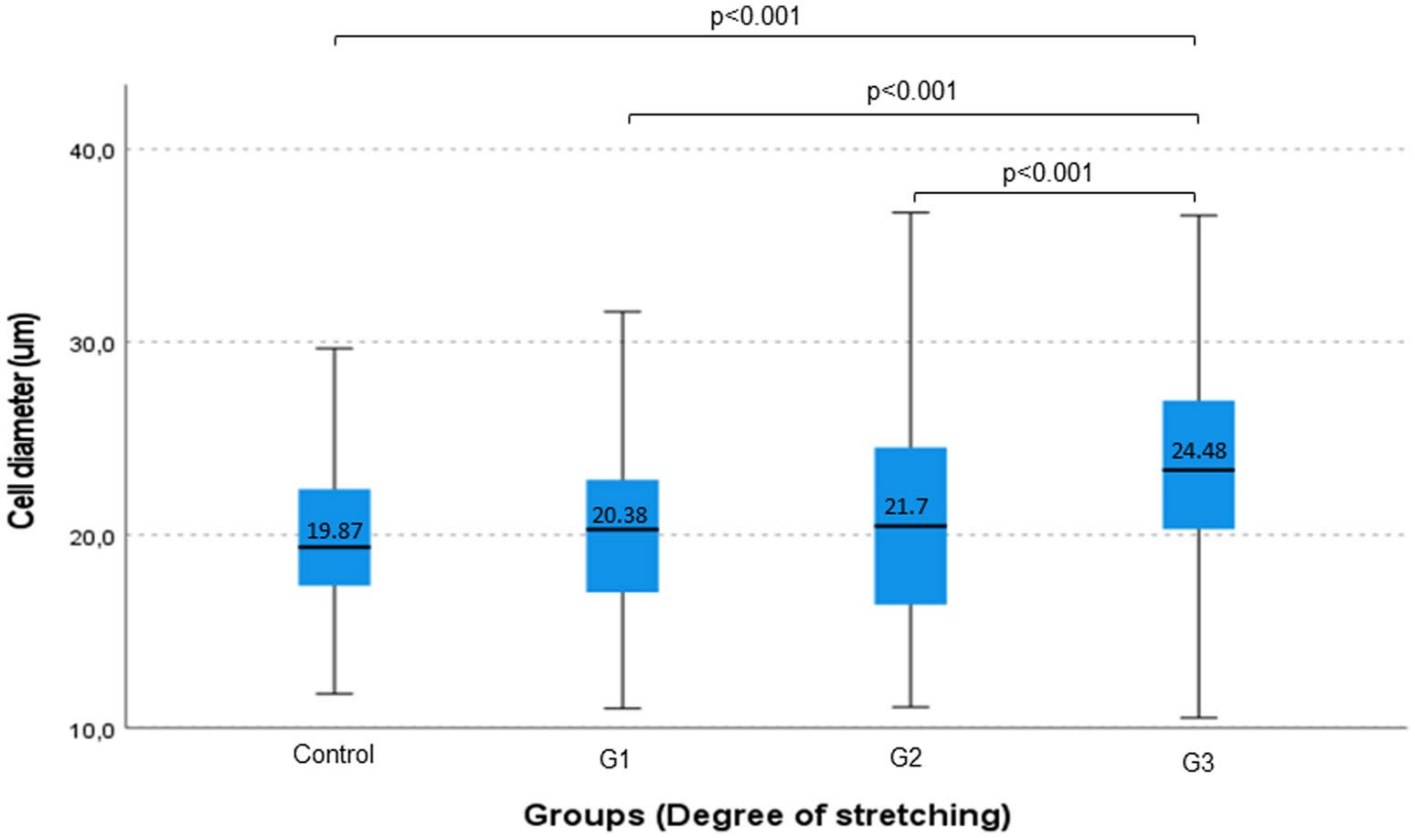
Diameter of the muscle cells. Boxplot diagram showing the measured cell diameters of the four Groups and the observed significant difference by paired comparisons of the Groups.

Normality test did not demonstrate normal distribution of measured data. Descriptive statistical analysis of measured cell diameters revealed raising mean and standard deviation among the groups (control μ = 19.87 μm, SD = 3.81; G1 μ = 20.38 μm, SD = 4.45; G2 μ = 21.7 μm, SD = 6.58; G3 μ = 24.48 μm, SD = 6.69). Ascending SD indicates growing variance of muscle cell diameters as a result of increasing stretch level. Diameter of the cells was not significantly different between control and G1 also control and G2. The most evident difference in muscle cell diameter was seen between the G3 and all the other groups (*p* < 0.001) Significance values were confirmed by the Bonferroni correction. (Fig. 7).

#### Distance between myofibrils

**Figure 8:**
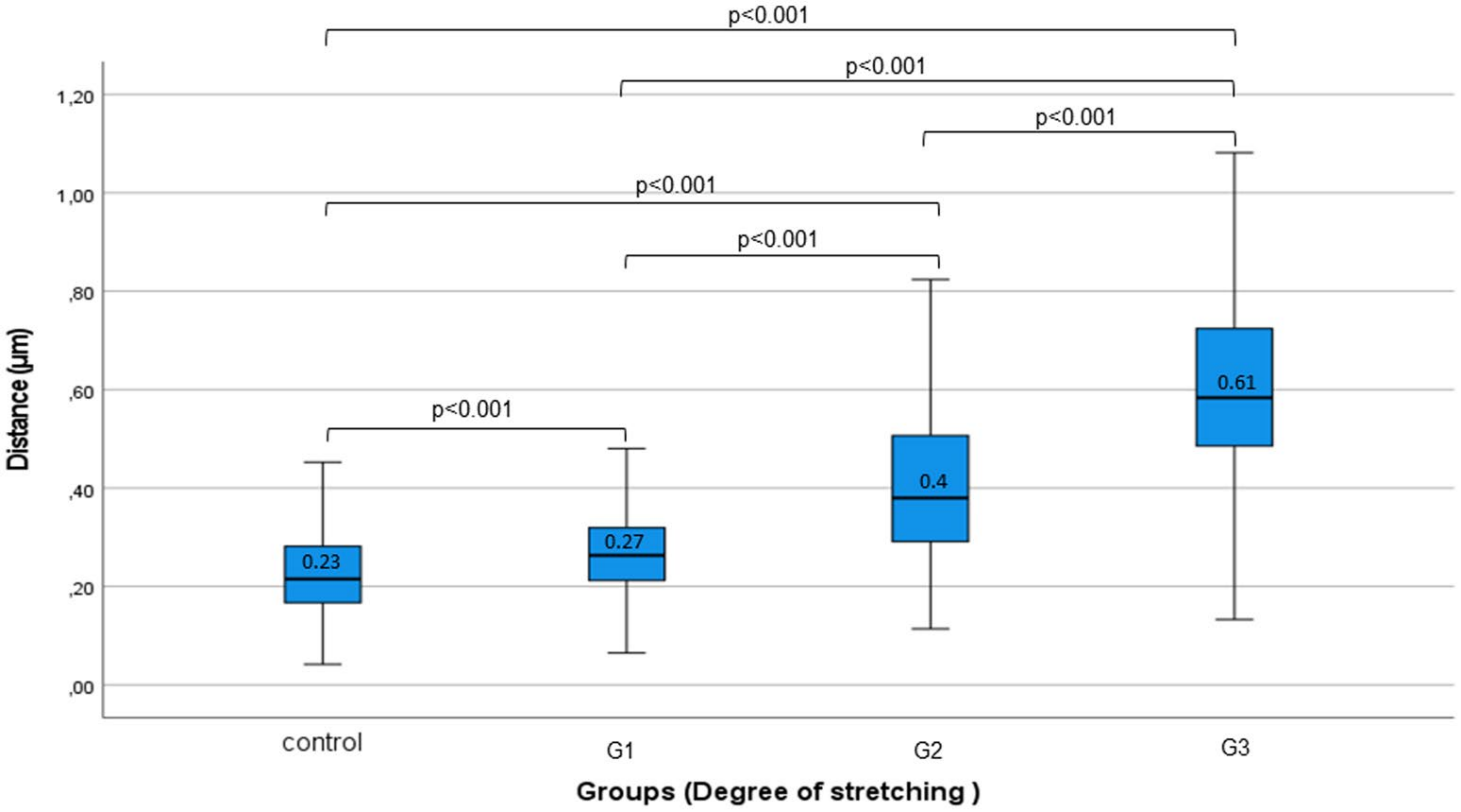
Distance between myofibrils. Boxplot diagram of measured distance between myofibrils. Paired comparisons of Groups demonstrated significant difference in between all of them.

Normality test showed not normal distribution of measured data. Results of descriptive statistical analysis are summarized as following: control μ = 0.23 μm, SD = 0.08; G1 μ = 0.27 μm, SD = 0.08; G2 μ = 0.4 μm, SD = 0.15; G3 μ = 0.61 μm, SD = 0.2. Statistical analysis showed significant difference in between all the groups as demonstrated on Fig. 8. Significance values were confirmed by the Bonferroni correction. Distance increases parallel to the applied stretch level. Ascending SD indicates growing variance of distance between myofibrils as a result of increasing stretch level.

## Discussion

With the recent interest in esophageal tissue engineering, designing of scaffold has raised the questions with regards to the stretching capability of these materials as esophagus is a structural as well as functional organ^15^. There is paucity of data on this topic as clinicians performing esophageal surgery have not assessed or quantified the stretch potentially of the esophagus in relation to the structural changes. While the quantification of the stretch potential was a subject of interest that was initiated to address mechanical demands of tissue engineered esophagus, this brought attention to the esophagus that was stretched as part of the repairs in esophageal atresia. Esophageal atresia repairs involve varying degrees of tension in order to achieve the anastomosis. Long-gap esophageal atresia staged repairs have further raised the questions on the changes in the microstructure of the esophagus that may influence clinical outcomes. The present in-vitro study was designed to investigate stretch related microscopic changes in the esophagus and to form a basis of quantification of stretch and the associated changes that these may cause in the surgical repair of esophagus.

This study concentrated on the effect of mechanical tension on ovine esophageal tissue, focusing on ultrastructural changes in *tunica muscularis* using uniaxial longitudinal stretching, which is a similar maneuver performed during esophageal atresia repairs. The length of an esophagus measured between upper and lower esophageal sphincter in a neonate is 8–10 cm^19, 22^. Long gap esophageal atresia (7–9% of esophageal atresia cases) are defined as a gaps more than 2 cm^20^, or 2 vertebral bodies^21^. Based on the data above, to bridge a 2 cm gap between the free ends of a newborn’s esophagus after complete dissection of the existing upper and lower pouches in single step is equivalent to a 25–20% stretch. Calculating with a 3 cm gap this elongation is 30–37.5% of the existing upper and lower esophageal ends. These data correlate with the observed significantly higher complication rate in long gap esophageal atresia repair^4^. Quantification of the degree of stretch and its correlation to ultrastructural changes in the esophagus is important as it will offer better insights into clinical outcomes.

In our previous study, we demonstrated that esophageal mucosal and submucosal layers withstand considerably high stretch levels, in contrast to muscular layer, which is damaged beyond 30% stretch^16^. The present study analyzed this data with significant comparative ultra-structural changes in esophageal tissue undergoing stretch. Muscle cells were separated due to stretch when compared to normal tissue, and the distance between muscle cells increased by advancing levels of stretch. Diameters of the muscle cells remain normal up to 30% stretching. Myofibrillar architecture was seen to be disrupted from 30 and 40% of stretch. As this is an in-vitro study, it would be difficult to speculate on the reorganization or functional changes in the esophagus due to these disruptive changes. However, it would be worthwhile to note these changes as it is clinically well known that esophageal atresia patients present with functional morbidities and swallowing associated conditions following repairs^4^. No study to date has correlated clinical measurements of esophageal stretch based on gap in esophageal atresia and long-term outcomes of repairs.

In long gap esophageal atresia repairs anastomosis is being achieved by staged procedures that employ stretching of the esophagus which is not quantified in terms of the % of stretch to the normal tissue. Structural changes after forceful elongation or stretching of tissues applied in surgical procedures for long gap esophageal atresia repair is still poorly understood. Human studies on mechanical esophageal stretching are not feasible due to the lack of equipment for performing such tests as well as the ethically unreasonable task of obtaining a full thickness esophageal biopsy at a later stage to analyze these tissues. Investigations on controlled esophageal elongation in animal models *in-vivo* will need to be designed in order to understand and demonstrate tissue alterations after considerable esophageal stretch and the potential of this tissue to reorganize in order to function properly or be subjected to permanent damage. These studies will require meticulous planning and maintenance of the experimental model for longer periods of defined times to understand the outcomes of tissue reorganization affected due to stretch.

The limitation of present study is small size samples (1–2 mm^3^ esophageal muscular layer samples) of tissues have been investigated by TEM and have focused mainly on the muscle aspect, whereas other factors affecting other sections of the esophagus could also be important; such as stretching of esophageal ganglions and disruptions of their connections. Although the present study has limitations, it has begun to unravel the subject of objectifying stretch and ultrastructural changes in the esophagus. Funding for studies using TEM is always a limiting factor as this investigation is associated with high costs when compared to light microscopy-based investigations. Further studies also need to be designed to investigate stretching effects on neuro-muscular junction and esophageal innervation. *In-vivo* studies with the esophagus are difficult to perform as the subject needs to be maintained on parenteral nutrition before feeds are commenced, which is quite challenging in the experimental step-up especially if large models are involved. In spite of all these, our study is an in vitro one, alive tissue and in vivo environment may act differently.

Our results draw attention to the possible presence of analogous ultrastructural changes in the human esophagus stretched up to 30% or more of its original lengths, which is a similar maneuver done in a newborn during one stage esophageal atresia repair with a gap equal to or bigger than 3 cm.

## Supplementary Information

Supplementary Legends.

Supplementary Table S1.

Supplementary Table S2.

Supplementary Table S3.

## Data Availability

All data generated and analyzed during this study are included in this published article [and its supplementary information files: Distance between muscle cells.xls, Distance between myofibrils.xls, Diameter of the muscle cells.xls].
