# Supplementary material for: Ultrastructural changes in esophageal tissue undergoing stretch tests with possible impact on tissue engineering and long gap esophageal repairs performed under tension
Source: Sci Rep. 2023 Jan 31;13:1750. doi: 10.1038/s41598-023-28894-5 (PMC9889733; doi:10.1038/s41598-023-28894-5)
Supplement: Supplementary file 1 — Supplementary Legends. [file 41598_2023_28894_MOESM1_ESM.docx]

**Legends for supplementary materials**

**1. Supplementary table 1 (Distance between muscle cells.xlsx) :** Column A indicates the Groups from 1 to 4 in which the measurements were done. Column B contains measurement data of the Distance between muscle cells metering in micrometers (μm)

**2. Supplementary table 2 (Distance between myofibrils.xlsx) :**Column A indicates the Groups from 1 to 4 in which the measurements were done.Column B contains measurement data of the Distance between myofibrils metering in micrometers (μm)

**3.  Supplementary table 3 (Diameter of the muscle cells.xlsx) :** Column A indicates the Groups from 1 to 4 in which the measurements were done.Column B contains measurement data of the Diameter of the muscle cells metering in micrometers (μm)

​
